# Stromme Syndrome: New Clinical Features

**DOI:** 10.21699/ajcr.v8i2.564

**Published:** 2017-03-18

**Authors:** Bayram Ali Dorum, Irmak Tanal Sambel, Hilal Ozkan, Irfan Kiristioglu, Nilgun Koksal

**Affiliations:** 1 Division of Neonatology, Department of Pediatrics, Uludag University Faculty of Medicine, Bursa; 2 Department of Pediatrics, Uludag University Faculty of Medicine, Bursa; 3 Department of Pediatric Surgery, Uludag University Faculty of Medicine, Bursa

**Dear Sir**

Stromme syndrome is extremely rare autosomal-recessive condition characterized by intestinal, ocular and cranial anomalies. In 1993 Stromme et al reported two sisters with jejunal atresia, cranial and ocular anomalies.[1] In 2007, van Bever et al first proposed the name as Stromme syndrome for patients with similar clinical conditions. [2]

A baby girl was born, on the 35th week of gestation via cesarean section, to an 18-year old mother. Apgar score at the 1st and 5th minute was 8 and 9, respectivly. Antenatal scan at 20th gestational week found microcephaly, edema in both lower extremities and the dilation of the proximal intestinal loops. No pathology was found on FISH examination in realtion to chromosome 13, 18, 21, X and Y during amniocentesis. At birth baby had weight of 1890 gram (10-50 percentile), the height of 40cm (<10 percentile) and head circumference of 26cm (<10 percentile). Examination of the head revealed microcephaly, micrognathia and a high-bridged nose (Fig. 1). Edema was seen in both lower extremities (Fig. 1). CBC showed thrombocytopenia (86.000/mm3). Liver and kidney function tests, and albumin level were in normal range. Serologic tests for TORCH and Parvovirus were negative. Abdominal ultrasonography (USG) showed bilateral renal hypodysplasia. Ventricular septal defect was found on Echocardiography. Ophthalmologic examination showed microphthalmia, microcornea, and sclerocornea.

**Figure:**
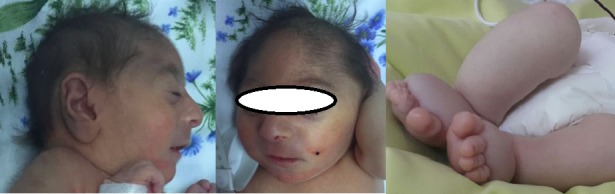
Figure 1: Patient had microcephaly, microphthalmia, micrognathia, and edema in both lower extremities.

On the second day, due to bilious vomiting, an abdominal radiograph was performed which showed double bubble appearance. On laparoscopic examination, Type 3a jejunal atresia was detected, resection and end-to-end anastomosis were performed. The cranial magnetic resonance imaging examination detected gyral simplification, cerebellar hypoplasia, and corpus callosum hypoplasia. Electroencephalogram examination was normal. With all these findings, the patient was diagnosed with Stromme syndrome. On the 28th day, the patient was discharged in good general condition.

Castori et al examined ten similar cases in the literature.[3] They found that jejunal atresia and ocular findings occurred in all patients however, the head circumference of three patients was normal. Eight of the patients were diagnosed with the apple peel type jejunal atresia. All of the patients had jejunal atresia. Amongst ocular findings, sclerocornea was most frequently seen. Other findings were microphthalmia, microcornea, ptosis, epicanthus, etc.[4] Unlike previous patients, edema in lower extremities was detected prenatally and was also seen at the birth in patient. Albumin level was normal. Edema was not observed in any other anatomical region. Edema gradually disappeared. Due to thrombocytopenia platelets were transfused before surgery. Thrombocytopenia also improved over time. To conclude, our paient was clinically diagnosed as Stromme syndrome. Unlike previous patients, this patient had additional features such as edema of lower extremities, renal parenchymal changes, thrombocytopenia, and cardiac anomaly.

## Footnotes

**Source of Support:** Nil

**Conflict of Interest:** None declared

